# The Protectin Family of Specialized Pro-resolving Mediators: Potent Immunoresolvents Enabling Innovative Approaches to Target Obesity and Diabetes

**DOI:** 10.3389/fphar.2018.01582

**Published:** 2019-01-17

**Authors:** Trond Vidar Hansen, Anders Vik, Charles N. Serhan

**Affiliations:** ^1^Department of Pharmaceutical Chemistry, School of Pharmacy, University of Oslo, Oslo, Norway; ^2^Department of Anesthesiology, Perioperative and Pain Medicine, Center for Experimental Therapeutics and Reperfusion Injury, Brigham and Women’s Hospital, Harvard Medical School, Harvard Institutes of Medicine, Boston, MA, United States

**Keywords:** protectins, specialized pro-resolving mediators, obesity, diabetes, G-protein coupled receptors, immunoresolvents, resolution pharmacology, western society diseases

## Abstract

A western type diet and lifestyle play an important role in the development of chronic diseases, yet little insight into the precise cellular and biomolecular mechanisms has emerged. It is known that an unbalanced diet may result in obesity and diabetes. Sufficient amounts and proper balance of omega-6 and omega-3 polyunsaturated fatty acids is key for maintenance of health. The resolution of inflammation is now held to be a biosynthetically actively driven process precisely regulated and controlled by a superfamily of specialized pro-resolving mediators. Specialized pro-resolving mediators are biosynthesized from both omega-6 and omega-3 polyunsaturated fatty acids and are resolution agonists acting on distinct G-coupled protein receptors. These mediators display potent anti-inflammatory and pro-resolving bioactions with EC_50_-values in the low nanomolar to picomolar range. The protectin (PD) family of specialized pro-resolving mediators is biosynthesized from the two omega-3 polyunsaturated fatty acids docosahexaenoic acid (DHA) and n–3 docosapentaenoic acid (n–3 DPA). All of the PDs display interesting bioactions as anti-inflammatory and pro-resolving agents. This review covers the bioactions, G-coupled protein receptors pharmacology, biosynthesis, and medicinal chemistry of the PD family of specialized pro-resolving mediators with an emphasis on obesity and anti-diabetic effects. In order to enable drug development and medicinal chemistry efforts against these diseases, stereoselective total organic synthesis of each of these mediators is required for confirmation of structure, stereochemical biosynthesis, and their functions. We provide an overview of our ongoing efforts and the current knowledge.

## Introduction

### Lipid Mediators in the Acute Inflammatory Response: Uncontrolled Inflammation and Neutrophil Responders

Obesity and diabetes are two highly prevalent pathological conditions of western society due to incorrect diet, tobacco use, alcohol consumption, and an increased sedative lifestyle (Leonard, 2008). Among these factors, the general medical opinion is that diet is a significant factor increasing incidence and mortality of these diseases (Leonard, 2008). An elevated intake of western type diet rich in red meat and processed food, i.e., a poor and pro-inflammatory diet, might develop into acute or chronic inflammation (Carrear-Bastos et al., 2011). This type of diet is high in omega-6 and rather low in omega-3 PUFAs (Simopoulos, 2006). Studies of the molecular, cellular and pharmacological processes involved in inflammation have revealed that such PUFAs are biosynthetically transformed to potent oxygenated lipid mediators that participate in the inflammatory processes (Cotran et al., 1999). The acute inflammatory responses are host-protective to contain foreign invaders and in health, are self-limited (Malawista et al., 2008). If uncontrolled, chronic inflammation may result in numerous diseases, including obesity and diabetes (Serhan, 2004). In both diseases, peripheral blood markers of inflammation are present in elevated levels after intake of a pro-inflammatory western type diet (Calder, 2017).

**FIGURE 1:**
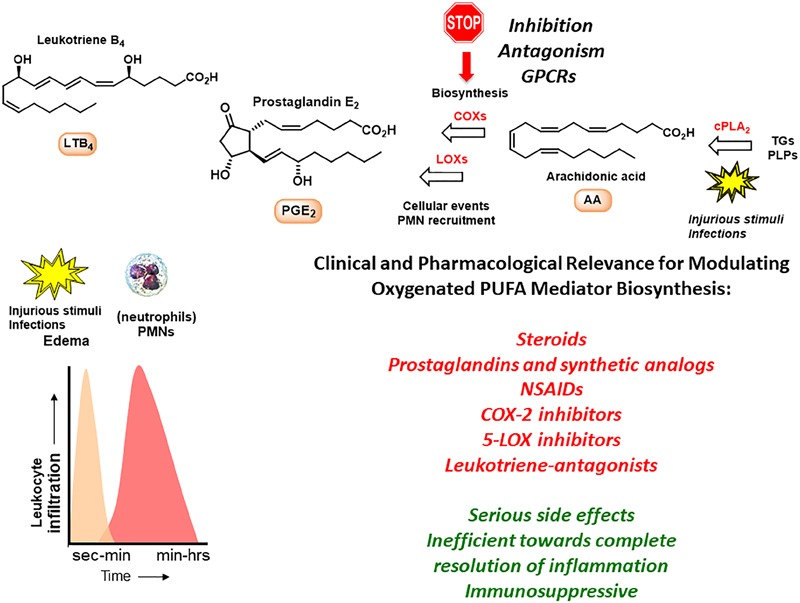
The inflammatory response and outline of biosynthetic pathways involving arachidonic acid. TGs, triglycerides; PLPs, phospolipids.

Studies led by Samuelson and co-workers on the biomolecular understanding of inflammation resulted in the identification of PGs, LTs and thromboxanes that act as pro-inflammatory mediators when formed in excess (Samuelsson, 2012). Examples are LTs B_4_ and C_4_ that are stereoselectively biosynthesized from the omega-6 PUFA AA in the presence of LOXs, while COXs form PGs (Samuelsson et al., 1987). AA is also involved in the biosynthesis of the anti-inflammatory and pro-resolving lipid mediators named LX A_4_ (LXA_4_) and LX B_4_ (LXB_4_) (Serhan, 1997). LTs, PGs and LXs act via individual GPCRs and play key roles in the early events and in the initiation of the inflammatory response by activating neutrophils (polymorphonuclear leukocytes, PMNs) (Samuelsson et al., 1987; Serhan, 1997). PMNs are the first cellular responders to the site of inflammation and aim to neutralize and clear foreign invaders. During the inflammatory response the biosynthesis of PGs and LTs occurs within seconds to minutes and increases with time (Figure 1; Samuelsson et al., 1987; Serhan, 1997; Buckley et al., 2014).

Several drugs have been developed that target chronic conditions and work toward dampening the effects from inflammatory markers. Steroids, such as cortisol, provided the first leads, but later the LTs and PGs have been used as lead compounds for the development of several anti-inflammatory drugs (Samuelsson, 2012). The LXs have also been the topic of drug discovery efforts (Petasis et al., 2005; Fetterman and Zdanowicz, 2009). Examples of anti-inflammatory drugs are the two non-selective COX-inhibitors ibuprofen and acetylsalicylic acid, the selective COX-2 inhibitor celecoxib, the leukotriene-antagonist montelukast and the 5-LOX inhibitor zileuton (Samuelsson, 2012).

### Beneficial Roles of Dietary Omega-3 Fatty Acids in Inflammatory Processes. Biosynthesis of Specialized Pro-resolving Mediators and Resolution

**FIGURE 2:**
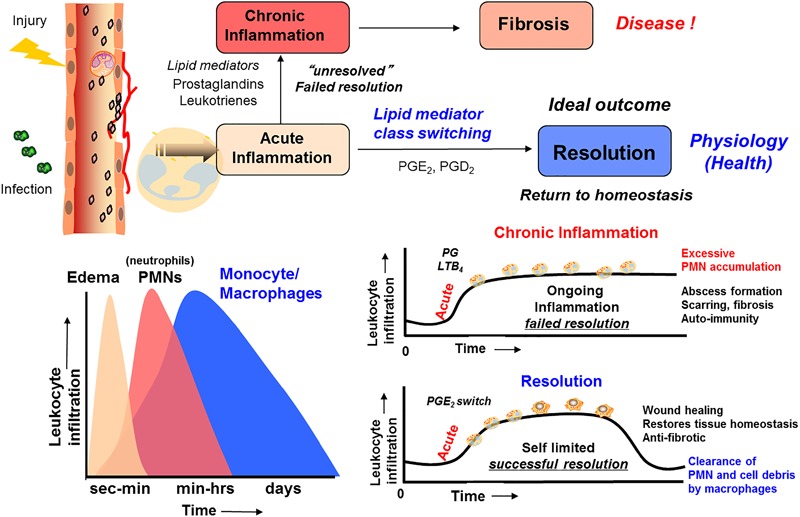
Outline of the *essential* lipid mediator *switch* involved in the return to homeostasis.

The omega-3 PUFAs EPA, n–3 DPA, and DHA, abundant in fatty fish and several dietary supplement products, have been attributed with several health benefits, including prevention of obesity and diabetes (Cotran et al., 1999). These PUFAs are essential as they are produced in only very limited amounts *de novo* in humans and thus must be obtained from our diets (Simopoulos, 2006; Calder, 2017). The cellular, pharmacological and biochemical modes of actions these PUFAs display in modulating these diseases are still under investigation. Of note, it was believed earlier that the host response was passive (Bannenberg et al., 2005; Serhan and Savill, 2005; Gordon, 2016) during resolution, and that eicosanoids (LT B_4_, PGs) (Bannenberg et al., 2005; Serhan and Savill, 2005), complement products (Ward, 2010) chemokines, and cytokines directed PMNs to local tissue sites (Medzhitov, 2015) with all of these mediators simply diluting over time within tissues (Figure 2). This dilution would then limit additional PMN recruitment and eventually enabling tissues to restore physiology (Bannenberg et al., 2005). However, numerous studies have shown that the LXs, biosynthesized from AA, function as potent and active stop signals for PMN influx characteristics of SPMs (Takano et al., 1998; Serhan et al., 2000) indicating that the resolution response is a biosynthetically active process (Serhan and Savill, 2005).

**FIGURE 3:**
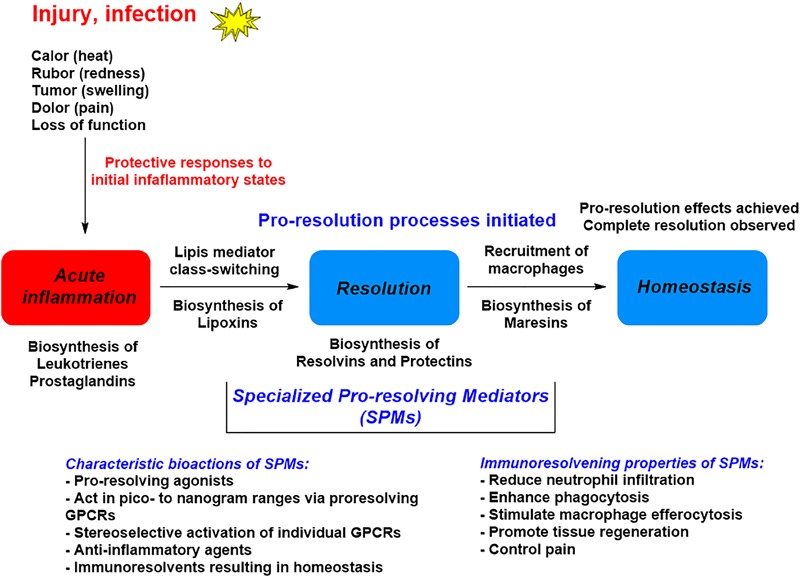
A general outline of the lipid mediator class switch involving SPM biosynthesis from EPA and DHA.

**FIGURE 4:**
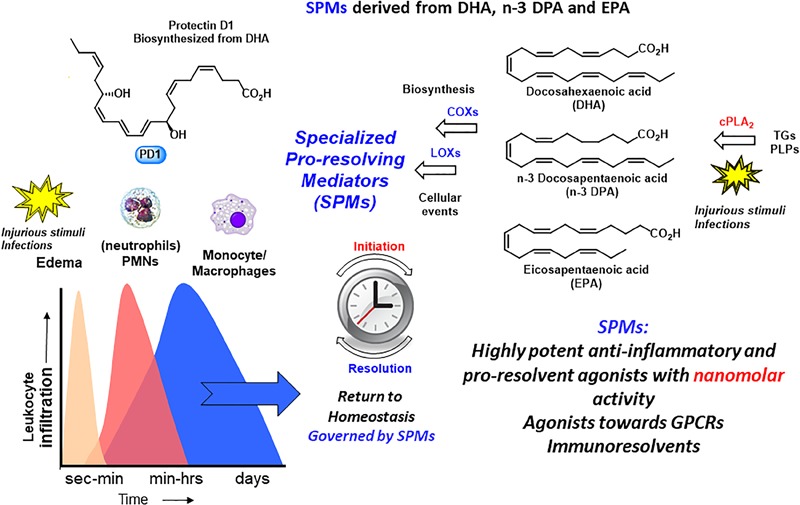
An outline of the biosynthetic formation of specialized pro-resolving mediators and their biosynthetic formation. TGs, triglycerides; PLPs, phospolipids.

The omega-3 PUFAs EPA and DHA, abundant in fat fish and used in dietary supplements, have been associated with many health benefits (Calder, 2017). SPMs may constitute the molecular basis for such positive claims in a wide range of clinical indications. Evidence has been provided over the last two decades on the detailed cellular and biochemical mechanisms showing that during self-limited inflammatory response a ***switch*** in the biosynthesis of pro-resolving SPMs occurs (Figure 2; Levy et al., 2001). This active biosynthesis increases with time. The switch in the biosynthesis of pro-resolving SPM autacoids provides a cellular, biochemical and detailed enzymatic mechanistic explanation on how the resolution of inflammation occurs and completes in order to regain a new homeostasis in contained inflammatory exudates (Figures 2, 3; Bannenberg et al., 2005). The molecular, biochemical and cellular events involved in the return to homeostasis have been coined catabasis (Serhan and Savill, 2005). For a schematic overview, please consult Figure 3. When the resolving secretory phospholipases cPLA2-IID and ZPLA2-III are stimulated (Takano et al., 1998) the PUFAs EPA, DHA and n–3 DPA are released from phospholipids enabling biosynthetic production of SPMs in specific organs (Figure 4; Levy et al., 2001). In exudates, unesterifed omega-3 PUFAs are delivered from blood via edema proteins for enzymatic conversion to SPMs (Kasuga et al., 2008), thus providing novel mechanisms for substrate availability for SPM biosynthesis to terminate further expansion of the cellular exudates (Murakami et al., 2015).

### Specialized Pro-resolving Mediators Are Resolution Agonists Acting on G-Protein Coupled Receptors

The SPMs display potent nanomolar agonist actions *in vivo*, that are stereoselective and act as ligands for individual GPCRs (Serhan and Chiang, 2013). The activation of one or several GPCRs induces cellular functions that carry out the potent bioactivities of the SPMs. Initial studies on the receptors for PD1 revealed cell-type specific activity that was also structure dependent (Levy et al., 2007). By using radiolabelled PD1 specific binding toward leukocytes was observed (Marcheselli et al., 2010). The identification that PD1 elicit signaling responses toward GPR37 was very recently reported by Bang et al. (2018). These investigators also reported that GPR37 activation in macrophages increased phagocytosis, altered cytokine release and promoted resolution of inflammatory pain. Table 1 lists the known GPCRs that SPMs activate to evoke resolution of inflammation *in vivo* in experimental animal models. Hence, it is possible that PD1 has additional receptors on neurons that directly regulate pain signaling.

**Table 1:** Reported receptors for specialized pro-resolving mediators.

Specialized pro-resolving mediator	Receptors	Human	Mouse
Lipoxin A_4_	ALX/FPR2; GPR32	Yes; Yes	Yes; n/a*
Resolvin E1	BLT1; CMKLR1; ERV	Yes; Yes	Yes; Yes
Resolvin D1	ALX/FPR2; GPR32; DRV1	Yes; Yes	Yes; n/a*
Resolvin D2	GPR18, DRV2	Yes; Yes	Yes; Yes
Resolvin D3	ALX/FPR2; GPR32; DRV1	Yes; Yes	Yes; Yes
Protectin D1 (Neuroprotectin D1)	GPR37, Pael-R	Yes	Yes

*^∗^Not available.*

## The Protectin Family of SPMs

**FIGURE 5:**
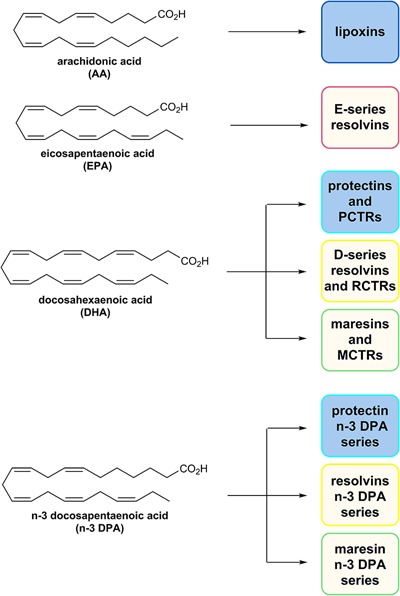
The chemical structures of AA, EPA, DHA, and n–3 DPA and an outline of the individual families of SPMs biosynthesized from these PUFAs.

As mentioned, the resolution of inflammation is now held to be a biosynthetically active process, regulated by biochemical mediators and receptor-signaling pathways governed by SPMs. The Serhan group employed lipid mediator proteomics, metabololipidomics (LC/MS-MS) and cell trafficking in self-limited inflammatory exudates to identify three new families of SPMs (Serhan et al., 2002, 2009; Hong et al., 2003; Dalli et al., 2013, 2014, 2015a,b; Ramon et al., 2016) coined the resolvins (*resolution phase interaction products*), PDs, and maresins (*macrophage mediators in resolving inflammation*). Each family is structurally distinct and biosynthesized from the n–3 essential fatty acids EPA, n–3 DPA, or DHA (Figure 5; Serhan et al., 2002, 2009; Hong et al., 2003; Dalli et al., 2013, 2014, 2015a,b; Ramon et al., 2016). PDs belong, together with the LXs, resolvins, maresin, as well as the sulfido-conjugates RCTRs, PCTRs, and MCTRs (maresin conjugates in tissue regeneration), to the super families of mediators (Figure 5). The PDs are structurally unique from the other SPMs because they possess a conjugated triene and their biosynthesis is initiated from the enzymatic production of a 17H*p*DHA intermediate.

### Pro-resolving and Anti-Inflammatory Actions of Protectins

**FIGURE 6:**
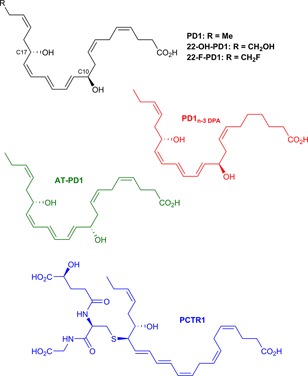
Chemical structures of PD1 and the epimer AT-PD1, PD1_n–3 DPA_, the metabolite 22-OH-PD1, the synthetic analog 22-F-PD1 and PCTR1. The chiral secondary alcohols are positioned at carbon atoms 10 and 17 for all protectins except PCTR1, while the primary alcohol in the metabolite 22-OH-PD1 is at carbon atom 22.

The protectin family of SPMs have attracted considerable interest from the biomedical community as resolution leads (Serhan, 2014; Fullerton and Gilroy, 2016; Dalli and Serhan, 2018). The precise cellular events, biochemical pathways and molecular mechanisms of the PDs in the resolution of inflammation is of interest in pharmacology and medicinal chemistry enabling drug development (Serhan, 2014; Fullerton and Gilroy, 2016; Dalli and Serhan, 2018). This SPM-subfamily is chemically characterized by two chiral secondary alcohols separated by an *E,E,Z*-triene moiety. Figure 6 depicts the distinct members of the PD family of SPMs.

The protectins are biosynthesized from DHA (Serhan et al., 2006; Aursnes et al., 2015) while the n–3 PDs are biosynthesized from n–3 DPA (Primdahl et al., 2017), with the individual biochemical steps presented in detail below. In addition to PMNs, PD1_n–3 DPA_ is produced by macrophages (Serhan et al., 2006; Dalli and Serhan, 2012) and eosinophils (Yamada et al., 2011; Katakura et al., 2015) and its production is reduced in severe asthma patients (Miyata et al., 2013). When PD1 is produced in neural systems, the name NPD1 is used to denote the location of the potent protective actions in retina, brain, and induction of pain (Bazan et al., 2010; Marcheselli et al., 2010; Asatryan and Bazan, 2017). Remarkable potent pro-resolving actions in mice with peritonitis was observed as only 1 ng of PD1 caused a reduction of PMN infiltration by approximately 40% (Serhan et al., 2006) PD1 display potent pro-resolution agonist effects with EC_50_ ∼ 1 nM and a K_d_–value of ∼31 pmol/mg of cell protein (Marcheselli et al., 2010).

### AT-PD1

The biosynthetic pathways mediated by human 15-LOX for the omega-6 AA derived LXs and their aspirin-triggered 15-epimeric forms are well established and studied (Serhan, 1997). PD1/NPD1 is biosynthesized predominantly in the 17*S* configuration by 15-LOX, but aspirin acetylation of COX-2 produces the hydroperoxide intermediate predominantly in the *R*-configuration at the 17-carbon position. This epimeric hydroperoxide is converted to the 17*R* epimer 17*R*-PD1, that is coined AT-PD1 (Serhan et al., 2002, 2011). The *R*-epimer is longer acting than the *S*-epimer PD1, most likely due to the stereochemical preference of the eicosanoid oxidoreductase enzymes for *S*-configured alcohols in the metabolism of oxygenated PUFAs (Serhan et al., 2011). AT-PD1 also display potent pro-resolving and anti-inflammatory actions as well as neuroprotective properties (Serhan et al., 2002, 2011).

### The PD1 Further Metabolite 22-OH-PD1 and the Synthetic Analog 22-F-PD1: Medicinal Chemistry Efforts

The further metabolism of PD1 once it is produced locally has not been studied *in vivo* in humans, but one study has reported a metabolite named 22-OH-PD1 (Figure 6) formed by ω-oxidation at the carbon atom number 22 (C-22) in PD1 (Serhan et al., 2002). This metabolite was prepared by total synthesis (Tungen et al., 2014). *In vivo* experiments in mice revealed that 22-OH-PD1 displayed potent pro-resolving and anti-inflammatory activities (Tungen et al., 2014). It is likely that additional further metabolic pathways of PD1 are mediated via eicosa oxidoreductases in the same way as for some of the other SPMs (Serhan and Petasis, 2011), although further studies are needed. The potent *in vivo* pro-resolution actions in the nanomolar range toward efferocytosis and phagocytosis that 22-OH-PD1 displayed spurred our interest in preparing the synthetic analog 22-F-PD1 depicted in Figure 6 (Tungen et al., 2018). When administered via intraperitoneal injection at 100 ng/mouse following *Escherichia coli* infection, 22-F-PD1 reduced PMN recruitment, enhanced macrophage phagocytosis and reduced bacterial load at similar levels to PD1 (Tungen et al., 2018). Overall, these results verified that the synthetic analog and putative medicinal chemistry agent 22-F-PD1 exhibited both potent anti-inflammatory and pro-resolving actions similar to native PD1. Macrophage phagocytosis and efferocytosis are both key pro-resolving biological actions of interest in drug discovery and clinical development (Serhan et al., 2002).

### The Novel Protectin PD1_n–3 DPA_

Recently, Dalli, Colas and Serhan in Boston, United States demonstrated that n–3 DPA is also a substrate for the biosynthesis of potent bioactive mediators that each correspond to the novel families of SPM (Dalli et al., 2013). While appreciated as an intermediate in omega-3 PUFA biosynthesis in humans, it is interesting that this PUFA is also a precursor to SPMs with 22 carbons and five double bonds (Dalli et al., 2013; Aursnes et al., 2014a). These n–3 immunoresolvents belong (Figure 5) to the three sub-families resolvins_n–3 DPA_, PD_n–3 DPA_, and maresin_n–3 DPA_ each demonstrating potent pro-resolving actions as identified in human subjects (Aursnes et al., 2014a; Markworth et al., 2016; Gobbetti et al., 2017; Hansen et al., 2017). To obtain further evidence for the complete structure of PD1_n–3 DPA_ it was essential to assess that the synthetic material carried the potent biological actions described for PD1_n–3 DPA_, see below for a discussion on its synthesis (Aursnes et al., 2014a). Administration of synthetic material using 10 ng per mouse significantly reduced neutrophil recruitment during peritonitis following zymosan A challenge (Aursnes et al., 2014a). These bioactions were comparable to those displayed by PD1. Moreover, synthetic material of PD1_n–3 DPA_ stimulated human macrophage phagocytosis and efferocytosis in the pico- to nanomolar range. Overall, these results verified that PD1_n–3 DPA_ exhibited both potent anti-inflammatory and pro-resolving actions, confirming the potent immunoresolvent properties of this SPM (Aursnes et al., 2014a). Potent protective bioactions for PD1_n–3 DPA_ and RvD5_n–3 DPA_ were demonstrated in mouse colitis and in reducing human PMN adhesion to endothelium (Gobbetti et al., 2017). In another recent study, PD1_n–3 DPA_ promotes resolution of neuroinflammation and arrests epileptogenesis potently due to a marked delay in the neuroinflammatory response (Frigerio et al., 2018). These studies were also the first to report that PD1_n–3 DPA_ regulates neuroinflammation (Frigerio et al., 2018). Recently Dalli and co-workers found that the biosynthetic pathway of the n–3 DPA PDs regulated the differentiation of human monocytes, altering macrophage phenotype, efferocytosis, and bacterial phagocytosis (Pistorius et al., 2018).

### Protectin Conjugates in Tissue Regeneration: PCTR1, PCTR2 and PCTR3

**FIGURE 7:**
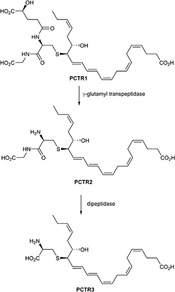
Enzymatic conversion of PCTR1 produces PCTR2 and PCTR3.

In 2014 and 2015 three new classes of SPMs were discovered and elucidated that carry potent tissue regenerative properties and posses anti-inflammatory and pro-resolving bioactions (Dalli et al., 2013, 2014). In Figure 6 the chemical structure of PCTR1 is depicted, and this novel SPM belongs to the novel peptide-conjugated PDs that contain a sulfido-bond at the carbon atom 16 and were identified from self-resolving *E. coli* infections in mice and in human spleen. The biosynthesis is presented below. Enzymatic conversion of PCTR1 in the presence of γ-glutamyl transpeptidase produces PCTR2 and dipeptidase actions yield PCTR3 (Figure 7; Dalli et al., 2014).

PCTR1 enhances resolution of infectious inflammation and is produced by human M2 macrophages (Dalli et al., 2015b). In addition, PCTR1 promoted human monocyte and macrophage migration potently and dose-dependently in the 0.001–10 nM range (Dalli et al., 2015b). Furthermore, PCTR1 increased macrophage and monocyte migration, enhanced macrophage efferocytosis, and accelerated tissue regeneration in planaria (Dalli et al., 2015b). It was also reported that PCTR1 is temporally regulated during self-resolving infection. At the peak of inflammation, PCTR1 enhanced macrophage recruitment and phagocytosis of *E. coli*, decreased PMN infiltration, and counter-regulated inflammation-initiating lipid mediators, including PGs (Dalli et al., 2015b). These findings demonstrated that PCTR1 is a potent monocyte- and macrophage-agonist regulating key anti-inflammatory and pro-resolving processes during bacterial infection.

## Protectins in Obesity and Diabetes

As mentioned, a pro-inflammatory western type diet results in an increased level of inflammatory cellular and biomolecular markers, including those biosynthesized from omega-6 and omega-3 PUFAs (Simopoulos, 2006). An expansion of adipose tissue mass associated with a low-grade type of inflammation has been observed with an excess intake of diet and nutrition. This chronic and unresolved inflammation of adipose tissue is harmful and may result in diabetes, insulin resistance and non-alcoholic fatty liver disease, all increasing maladies in western societies (Clària et al., 2017). White adipose tissue plays essential roles in balancing metabolic and energy homeostasis (Clària et al., 2017). This balance is affected by AA, EPA, and DHA. In this setting, Clària and co-workers reported the first investigations on SPM biosynthesis in white adipose tissues given elevated levels of EPA and DHA (Gonzales-Periz et al., 2006, 2009; Clària et al., 2017). These studies showed that dietary amplification of DHA results in increased biosynthesis of PD1 and its precursor 17*S*-H*p*DHA (Gonzales-Periz et al., 2006), enzymatically reduced *in vivo* to 17*S*-HDHA (Serhan and Petasis, 2011). Using a transactivation assay, 17*S*-HDHA was shown to be a PPARγ-agonist (Gonzales-Periz et al., 2006). This finding is of interest since several PPARγ-agonists, such as the glitazones, have been developed as anti-diabetic drugs (Gonzales-Periz et al., 2006; Clària et al., 2017). Clària and co-workers also demonstrated that administration of DHA diminished the presence of pro-inflammatory PGs and LT B_4_ (LTB_4_) (Gonzales-Periz et al., 2006). Synthetic 17-HDHA stopped genotoxic and oxidative damage in hepatocytes and diminished 5-LOX expression in macrophages. In further studies, these authors reported that the biosynthetic formation of SPMs was severely deregulated in inflamed white adipose tissues as well as in obese mice (Gonzales-Periz et al., 2009). PD1 and RvD1 were reported to be the dominant DHA-derived SPMs based on LC/MS-MS metabololipidomic analyses. They also reported that reduced insulin resistance was observed in white adipose tissues, observations that were in parallel with initiation of phosphorylation of adenosine monophosphate and adiponectin, important regulators of systemic energy balance (Gonzales-Periz et al., 2009).

A skewed biosynthetic process was also observed when these investigators used white adipose tissue from patients with peripheral vascular diseases. In these patients the inflammatory status of white adipose tissue is severely altered (Clària et al., 2013). Within the setting of obesity and diabetes these observations could be due to the diminished tissue levels of omega-3 PUFAs since it has been reported that an increased intake of omega-3 PUFAs enhance SPM biosynthesis (Mas et al., 2012). The reduced level of SPMs quantified could also be explained by an enhanced catabolism or metabolism followed by conversion to further inactive metabolites of SPMs. Interestingly, in obese adipose tissue the enzyme eicosanoid oxidoreductase (15-PG-dehydrogenase) is markedly up-regulated (Gonzales-Periz et al., 2009). This enzyme is involved in the metabolic formation of 17-oxo-RvD1 and 7-oxo-RvD2 from RvD1 and RvD2 (Serhan and Petasis, 2011), respectively, that also occurs in white adipose tissue (Clària et al., 2012). The enzyme soluble epoxide hydrolase 2 (sEH) converts epoxides (Haeggström and Funk, 2011; Serhan and Petasis, 2011), some of which are intermediates in PD biosynthesis, formed from EPA and DHA, into diols with lower pro-resolving and anti-inflammatory properties (Haeggström and Funk, 2011; Serhan and Petasis, 2011). The enzyme sEH is found invariably overexpressed in obese mice (Lopez-Vicario et al., 2015). Overall, the studies from Clària et al. (2017) showed that an unbalanced level of SPMs are directly connected to insufficient tissue resolution in both *in vitro* and *in vivo* models of diabetes and obesity.

Of interest, Kuda et al. (2016) reported the isolation and characterization of new DHA-derived fatty acid esters of hydroxy fatty acids present in both serum and white adipose tissue after supplementation with DHA. LC/MS-MS results supported the assigned structures without information on the absolute configurations of the compounds. They performed experiments using mice as well as serum from obese patient with diabetes. The novel compound named 13-DHAHLA showed anti-inflammatory properties at much higher concentrations than SPMs (Kuda et al., 2016). These authors also found that 13-DHAHLA hindered the increase in several pro-inflammatory markers, such as interleukin-6, tumor necrosis factor-α, and PGs. In addition, 13-DHAHLA enhanced phagocytosis in zymosan A induced in an *in vitro* bone marrow derived macrophage assay (Kuda et al., 2016). For the assignment of absolute configuration of these branched DHA esters of hydroxyl substituted fatty acids, stereoselective total synthesis will be required. With synthetic material in hand, investigations toward which GPCR(s) these novel compounds activate, but also thorough *in vivo* experiments can be performed toward elucidation of any pro-resolving and anti-inflammatory activities these compounds may display.

**FIGURE 8:**
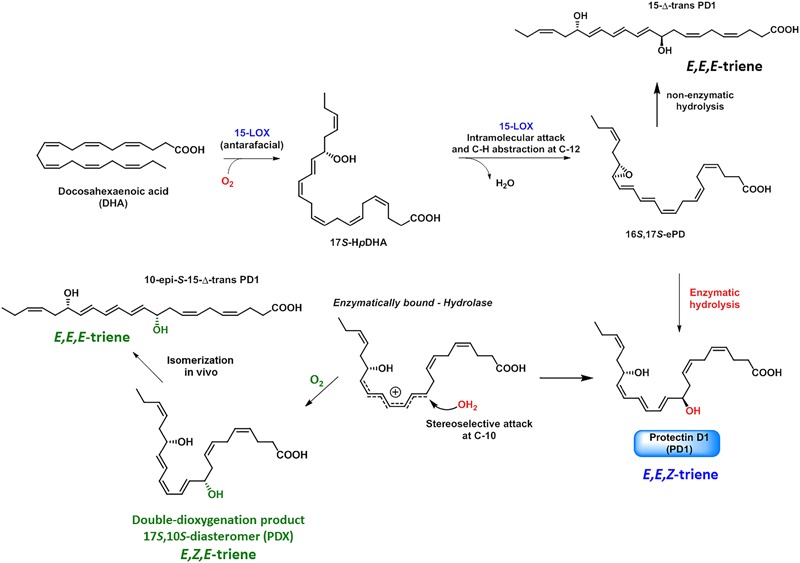
Biosynthesis of PD1, PDX, and isomers formed *in vivo*.

An isomer of PD1, named protectin DX (PDX), see below and Figure 8 for structure, has been confirmed formed and isolated from white adipose tissue (White et al., 2014). This DHA-derived compound has been reported to alleviate insulin resistance in db/db mice (White et al., 2014, 2015). Of note, PDX did not resolve white adipose tissue inflammation (White et al., 2014). However, *in vivo* studies with mice showed that both PD1 and PDX were able to modulate PPARγ transcriptional activity (White et al., 2015).

## Structural Elucidation and Determination of Absolute Configuration Using Biosynthetic and Synthetic Studies

In order to elucidate the complete structure of PD1 the correct absolute configuration and olefin geometry had to be determined for this potent SPM. Efforts from the Serhan research team on the biosynthesis of PD1 allowed the complete structural assignment of PD1 (Serhan et al., 2006). Experiments using isotopic oxygen incorporation and acid alcohol trapping products provided LC/MS-MS data that supported the involvement of an epoxide intermediate (Figure 8; Serhan et al., 2006).

The detailed and stepwise biosynthesis of PD1 has now been established as depicted in Figure 8 (Serhan et al., 2006; Aursnes et al., 2015). The enzyme 15-LOX type I functions as a 17-lipoxygenase (Haeggström and Funk, 2011) and forms the 16*S*,17*S*-configured epoxide named 16*S*,17*S*-ePD. Hydrolysis of this epoxide in a regio- and stereoselective manner at the C-10 position results in the formation PD1. Water attack occurs most likely via a transient allylic carbocation specie, see Figure 8, since the thermodynamically less stable 11*E*,13*E*,15*Z*-configured triene is formed, and not the chemically more stable 11*E*,13*E*,15*E* triene. Moreover, the *S*-configuration at C17 is not altered (Serhan et al., 2006; Aursnes et al., 2015). The formation of the 17*R*-H*p*DHA stereoisomer has been observed in the presence of aspirin and recombinant isolated COX-2 enzyme (Serhan et al., 2002, 2011), that results in the formation of the 17*R*-epimer of PD1, coined aspirin triggered protectin D1 (AT-PD1). This biosynthetic pathway occurs most likely via a 16*R*,17*R*-configurated epoxide intermediate named ePD. Later direct evidence that 16*S*,17*S*-ePD is in fact the true intermediate in the biosynthesis of PD1 was provided by Serhan, Hansen and co-workers (Aursnes et al., 2015). These biosynthesis studies were performed using 16*S*,17*S*-ePD, stereoselectively prepared by organic synthesis, that confirmed that this epoxide was converted into PD1 in human macrophages (Aursnes et al., 2015). The double LOX product 10,17-diHDHA, named PDX, is biosynthetically produced by two sequential oxygen insertion steps followed by reduction of the 15-LOX produced hydroperoxide-intermediate, affords 10*S*,17*S*-diHDHA (Serhan et al., 2006). PDX is an isomer of PD1 and has several reported bioactions relevant for diabetes and obesity (White et al., 2014, 2015). PDX has also been subjected to other biological investigations (Masterson et al., 2015; Stein et al., 2016; Fonseca et al., 2017; Körner et al., 2018). The other isomers of PD1 investigated were reported to possess significant lower potent pro-resolving actions (Serhan et al., 2006). It has recently been demonstrated that PCTR1 is also biosynthesized directly from 16*S*,17*S*-ePD (Ramon et al., 2016). Regarding the biosynthesis of the congener PD1_n–3 DPA_ the epoxide named 16*S*,17*S*-ePD_n–3 DPA_ has been shown to be an essential biosynthetic intermediate involved in the formation of PD1_n–3 DPA_ in human neutrophils (Primdahl et al., 2017). This epoxide is able to inhibit human neutrophils LTB_4_ production and that an yet unidentified hydrolytic enzyme converts 16*S*,17*S*-ePD_n–3 DPA_ into PD1_n–3 DPA_ (Primdahl et al., 2017).

## Reported Total Synthesis of PD1

As of today, the exact structural elucidation of SPMs using LC/MS-MS based metabololipidomics is necessary to establish the exact structure of the endogenously formed bioactive products (Chiang and Serhan, 2017), since only pico- to nanogram amounts of biosynthetic material are formed *in vivo*. Total syntheses of PD1 have been reported by four research groups (Ogawa and Kobayashi, 2011; Petasis et al., 2012; Aursnes et al., 2014b; Rodriguez and Spur, 2014), but only two groups have used synthetic and authentic material for matching experiments using LC/MS-MS multiple reaction monitoring (MRM). We want to emphasize that such efforts are of vitally importance, since the PDs as well as the other SPMs, display very potent agonist actions toward individual GPCRs in a stereochemically defined manner (Serhan and Petasis, 2011; Chiang and Serhan, 2017). The structural elucidation and physiologic functions of the SPM receptors have recently been reviewed (Chiang and Serhan, 2017). An outline of the different total syntheses of PD1 is presented below.

### Kobayashi and Ogawa’s Synthesis of PD1

**FIGURE 9:**
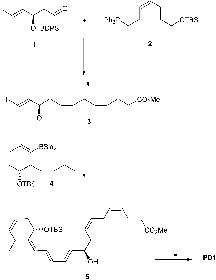
Outline of Kobayashi and Ogawa’s synthesis of PD1.

Ogawa and Kobayashi (2011) disclosed the first total organic synthesis of PD1, which included a Z-selective Wittig reaction and a Suzuki-cross coupling as key steps (Figure 9). These authors prepared the iodide-aldehyde fragment **1** in several steps and then reacted **1** with the ylide of **2** in a *Z*-selective Wittig reaction to afford, after several other synthetic steps, the ester **3**. This ester was subjected to a Suzuki-Miyaura cross-coupling reaction with boron-compound **4** affording compound **5** with the C22 carbon skeleton of PD1 in place. Deprotection of **5** was followed by basic hydrolysis that yielded PD1 (Ogawa and Kobayashi, 2011).

### The Petasis Synthesis of PD1

**FIGURE 10:**
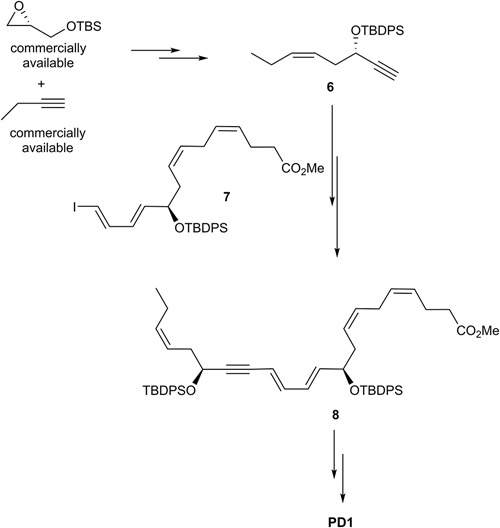
Outline of Petasis and co-workers synthesis of PD1.

The first synthesis and assignment of PD1 was reported by Serhan et al. (2006). These efforts also provided synthetic stereoisomers. Biological evaluations of PD1 and its synthetic stereoisomers provided useful information on structure-functions. For the stereochemical assignment Petasis et al. (2012) reported their synthesis in details in 2012, although synthetic PD1 as well as isomers were made available for biological studies from this group much earlier (Serhan et al., 2006). The Petasis synthesis utilized a Sonogashira cross coupling reaction to make the C22 carbon skeleton (Figure 10). The precursor **6** was reacted with **7** using this cross-coupling reaction to yield **8**. Of note, the commercially available starting material *R*-glycidol gave rise to both **6** and **7** (Petasis et al., 2012).

In order to obtain a Z-selective reduction of the conjugated alkyne **8** and to establish the correct *Z*-geometry of the double bond at C11-C12 (Serhan et al., 2006; Petasis et al., 2012), deprotection of the TBS-groups was first performed, thenthe authors used the Boland reduction reaction on **8** that was followed by basic hydrolysis, which furnished PD1. In addition, several isomers of PD1 were synthetized by the same research group. These isomers proved to be less potent pro-resolvents; however these studies provided useful information on structure-function relationships of PD1. The synthetic material was matched with endogenously formed PD1 and found to be identical (Serhan et al., 2006; Petasis et al., 2012).

### The Hansen Synthesis of PD1

**FIGURE 11:**
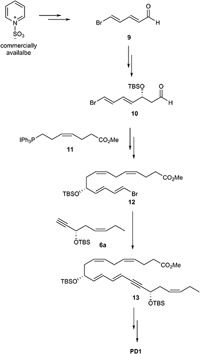
Outline of Hansen and co-workers synthesis of PD1.

A highly stereoselective synthesis of PD1 was published in 2014 (Aursnes et al., 2014b), mainly by using Evans-Nagao aldol-, *Z-*selective Wittig-, and Sonogashira-reactions (Figure 11). The main fragments were the terminal alkyne **6a**, the aldehyde **9** and the Wittig-salt **11**. The total synthesis of PD1 was performed in only eight linear steps from aldehyde **9**. Commercially available pyridinum-1-sulfonate was used for making aldehyde **9**. Further on, the aldehyde **9** was reacted in an Evans-Nagao aldol reaction that was followed by protection of the secondary alcohol and removal of the auxiliary to afford the intermediate **10** with high stereoselectivity. Then compound **10** was reacted with the corresponding ylide of the Wittig-salt **11** to yield the tetraene ester **12**. The alkyne **6a** was reacted with **12** in a Sonogashira cross-coupling reaction to afford **13** with the whole carbon skeleton of PD1. Deprotection of **13** and a Z-selective Lindlar reduction gave the correct *Z*-geometry of double bond at C15-C16. Saponification and acidic work-up furnished PD1. The synthetic material was matched with endogenously formed PD1 and found to be identical and with high chemical purity and stereochemical integrity (Aursnes et al., 2014b). The difference between alkyne **6**, used by the Petasis-group, and alkyne **6a** is the protection group.

### Spur and Rodriguez Synthesis of PD1

**FIGURE 12:**
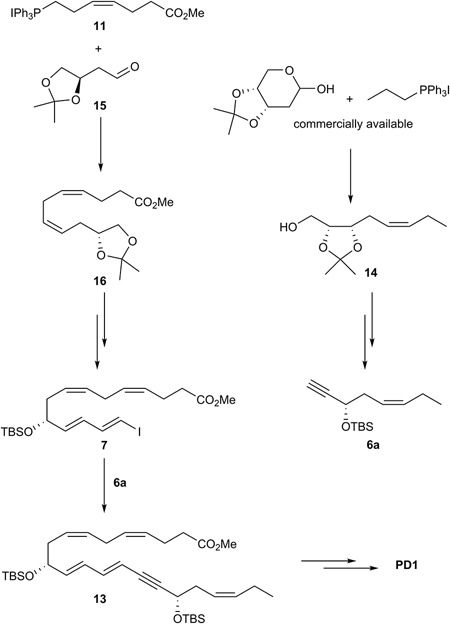
Outline of Spur and Rodriguez’ synthesis of PD1.

Rodriguez and Spur (2014) reported their total synthesis of PD1 that also relied on the Sonogashira cross-coupling reaction with the terminal alkyne **6a** (Figure 12). Conversion of commercially available acetal-protected D*-*ribose gave **14** that was converted into alkyne **6a** using a different synthetic route than reported by Petasis and co-workers. The ylide of Wittig salt **11** was reacted with the aldehyde **15** in a Z-selective Wittig-reaction to give intermediate **16**. This intermediate was transformed into the vinylic iodide **7** (Figure 12). Finally, fragments **6a** and **7** were reacted using the Sonogashira reaction to give **13** that completed the C22 carbon-chain of PD1. The last steps included deprotection of **13** and a *Z*-selective alkyne reduction using the Boland protocol as well as an ester hydrolysis that gave PD1 (Rodriguez and Spur, 2014).

### Summary and Future Directions for SPM Therapy in Obesity and Diabetes

The cardinal signs of inflammation: *calor, rubor, tumor, dolor*, and *functio laesa* are physiologically mediated by chemical mediators, such as the PGs, and are effectively controlled by traditional NSAIDs (Vane and Botting, 2001). These drugs give unwanted side effects. Given the increase in the inflammation-associated diseases obesity and diabetes it is paramount that new treatments and mechanisms are sought to control excessive inflammation and collateral tissue damage created by excessive PMN and their swarming in all organs (Cotran et al., 1999). Evidence for active endogenous resolution programs and novel resolution mediators holds promise for new therapeutic approaches that would not be immunosuppressive, but rather serve as immunoresolvents and pro-resolving mediator agonists stimulating resolution (Morris et al., 2009; Serhan, 2014; Perretti, 2015; Serhan et al., 2015; Duvall and Levy, 2016; Fullerton and Gilroy, 2016; Serhan, 2017; Vik et al., 2017; Dalli and Serhan, 2018). While current treatments for inflammation can be effective, many of these can eventually become immunosuppressive, opening opportunities for infection.

The distinct properties of EPA, DHA and n–3 DPA exhibits to form pro-resolving lipid mediators may, at least in part, explain the established health effects associated with these omega-3 PUFAs. The biosynthetic pathways of these potent lipid mediators may also explain some of the positive effects of aspirin, since COX-2 in the presence of aspirin biosynthesize metabolically longer lasting epimers of the individual SPM (Serhan and Petasis, 2011; Serhan et al., 2011). These epimers also display potent pro-resolving and anti-inflammatory properties. The different families of SPMs display high structural complexity due to the presence of several stereogenic centers, both in the form of chiral, secondary alcohols and conjugated *E*- and *Z*-double bonds, reflecting their biochemical origins, functions and stereospecific bioactions toward individual GPCRs. Hence, acquired knowledge and distinct care must be exercised when working with SPMs. Failure of such diligent operations will not reveal the correct and exciting chemical structures or the potent bioactions that SPMs possesses as resolution agonists. Elucidating the role of PUFAs as precursors and their enzymatic oxygenated products at the cellular and molecular level in health is of current interest (Serhan, 2017). As of today, approximately 80 biologically active DHA-derived metabolites have been described with various biological roles. It is important to emphasize that as of today only the endogenous SPMs, such as the PDs, display both potent pro-resolving and anti-inflammatory bioactions *in vivo* in the low nanomolar range. Hence, the PDs are therefore among the most exciting small molecules currently under investigations toward drug development based on resolution of inflammation (Gilroy et al., 2004; Morris et al., 2009; Serhan and Petasis, 2011; Tabas and Glass, 2013; Corminboeuf and Leroy, 2014; Perretti, 2015; Serhan et al., 2015; Duvall and Levy, 2016; Serhan, 2017; Vik et al., 2017). PDs display high structural complexity due to the presence of several stereogenic centers, both in the form of chiral, secondary alcohols and conjugated *E*- and *Z*-double bonds reflecting their biochemical origins, functions and stereospecific bioactions toward individual GPCRs (Duvall and Levy, 2016). Hence, care must be exercised when working with these SPMs as resolution agonists or as pharmacological biotemplates toward drug development targeting diabetes and obesity (Tabas and Glass, 2013).

The vast majority of approved drugs have been developed to inhibit, block or antagonize specific biological pathways involved in inflammatory conditions (Vane and Botting, 2001). Hence, the inflammatory mechanisms have become central to several diseases, including obesity and diabetes. The detailed biochemical, genetic, molecular, and cellular mechanisms behind the biology of resolution of inflammation has resulted in a new paradigm in our understanding of the inflammatory process. With the appreciation and growing understanding of these intervening mechanisms drugs within “Resolution Pharmacology” will be of interest. Examples of such candidates may be synthetic small molecular mimetics (Corminboeuf and Leroy, 2014; Vik et al., 2017) exhibiting pro-resolution and anti-inflammatory GPCR agonistic properties against obesity and diabetes (Oh and Olefsky, 2016). Moreover, activators of SPM biosynthesis or inhibitors of eicosanoid oxidoreductase (15-PG-dehydrogenase) may also be part of the potential future within the “Resolution Pharmacology” pharmacopeia. Of significance, SPMs are very potent GPCR agonists and approximately 40% of all approved drugs activate this receptor class. Based on the drug development efforts that pro-inflammatory PGs and LTs have resulted in, combined with an increasingly number of receptors identified, future drug development efforts should be facilitated (Gilroy et al., 2004; Tabas and Glass, 2013; Corminboeuf and Leroy, 2014; Duvall and Levy, 2016; Vik et al., 2017). However, such future endeavors depends, similar to past drug development successes based on pro-inflammatory mediators and their biological roles (Samuelsson, 2012), on basic biomedical research. We envision that the development of “Resolution Pharmacology” as well as exciting new findings from basic research related to SPMs will continue to evolve and enable innovative approaches for treating obesity and diabetes.

## Author Contributions

All authors contributed to the writing of the manuscript and had gave approval to the final version of the manuscript.

## Conflict of Interest Statement

CS has filed patents on SPMs and related compounds, licensed for clinical development, and his interests are reviewed and managed by BWH and Partners HealthCare in accordance with their conflict of interest policies. The remaining authors declare that the research was conducted in the absence of any commercial or financial relationships that could be construed as a potential conflict of interest. The reviewer HA declared a past co-authorship with one of the authors to the handling Editor.

## Abbreviations

AA: arachidonic acid
COX: cyclooxygenase
DHA: docosahexaenoic acid
n–3 DPA: docosapentaenoic acid
EPA: eicosapentaenoic acid
ePD: epoxide biosynthesized from DHA and an intermediate in protectin D1 biosynthesis
ePD_n–3 DPA_: epoxide biosynthesized from n–3 DPA and an intermediate in protectin D1 biosynthesis
GPCR: G-protein coupled receptor
LC/MS-MS: liquid chromatography tandem mass spectrometry
LM: lipid-derived mediators
LOX: lipoxygenase
LT: leukotriene
LX: lipoxin
LXA_4_: lipoxin A_4_ (5*S*, 6*R*, 15*S-*trihydroxy-eicosa-7*E*, 9*E*, 11*Z*, 13*E*-tetraenoic acid)
LXB_4_: lipoxin B_4_: (5*S*, 14*R*, 15*S-*trihydroxy-eicosa-6*E*, 8*Z*, 10*E*, 12*E*-tetraenoic acid)
Maresin: macrophage-derived resolution mediator of inflammation
MaR1: maresin 1 (7*R*, 14*S*-dihydroxy-docosa-4*Z*, 8*E*, 10*E*, 12*Z*, 16*Z*, 19*Z*-hexaenoic acid)
MCTRs: maresin conjugates in tissue regeneration
PCTR: protectin conjugates in tissue regeneration
PD: protectin
PD1: protectin D1 (10*R*, 17*S*-dihydroxy-docosa-4*Z*, 7*Z*, 11*E*, 13*E*, 15*Z*, 19*Z*-hexaenoic acid), also named neuroprotectin D1 (NPD1)
PD1_n–3 DPA_: PD1_n–3 DPA_ biosynthesized from n–3 DPA (10*R*, 17*S*-dihydroxy-docosa-7*Z*, 11*E*, 13*E*, 15*Z*, 19*Z*-pentaenoic acid)
PG: prostaglandin
PLP: phospolipid
PMN: polymorphonuclear leukocyte
PUFA: polyunsaturated fatty acid
RCTRs: resolvin conjugates in tissue regeneration
Rv: resolvin, bioactive omega-3 derived resolution phase interaction products
sEH: soluble epoxide hydrolase
SPM: specialized pro-resolving mediators
TG: triglyceride
